# A Rare Co-occurrence of Duchenne Muscular Dystrophy and Glycerol Kinase Deficiency Associated With Xp21 Contiguous Gene Deletion Syndrome: A Case Report

**DOI:** 10.7759/cureus.100053

**Published:** 2025-12-25

**Authors:** Lúcia Marques, Patrícia Lipari Pinto, Joana Coelho, Cristina Florindo, Mariana Soeiro e Sá, Raquel Rodrigues, Ana Sousa, Ana Gaspar

**Affiliations:** 1 Department of Pediatrics, Hospital Beatriz Ângelo, Lisbon, PRT; 2 Hereditary Metabolic Disease Reference Center, Metabolic Unit, Department of Pediatrics, Santa Maria’s Hospital, Lisbon North University Hospital Center, Entidade Pública Empresarial (EPE) Faculty of Medicine, University of Lisbon, Lisbon, PRT; 3 Neuropediatric Unit, Department of Pediatrics, Santa Maria’s Hospital, Lisbon North University Hospital Center, Entidade Pública Empresarial (EPE) Faculty of Medicine, University of Lisbon, Lisbon, PRT; 4 Laboratory of Metabolism and Genetics, Department of Pharmaceutical Sciences and Medicines, Faculty of Pharmacy, University of Lisbon, Lisbon, PRT; 5 Department of Medical Genetics, Santa Maria’s Hospital, Lisbon North University Hospital Center, Entidade Pública Empresarial (EPE) Faculty of Medicine, University of Lisbon, Lisbon, PRT

**Keywords:** array-cgh, complex glycerol kinase deficiency, contiguous gene deletion syndrome, duchenne muscular dystrophy, intellectual disability

## Abstract

Glycerol kinase deficiency is an X-linked disorder and can occur in isolation or combined as part of the Xp21 continuous gene deletion syndrome. We report the first offspring of a non-consanguineous couple with this contiguous gene deletion syndrome. There was a family history of short stature and learning difficulties, and a personal history of two hospitalizations due to prostration, hypoglycemia, and metabolic acidosis, the first of which occurred at six months of age. The patient was referred to a neurodevelopment consultation due to a global developmental delay detected at two years of age and a genetic consultation at four years old. The array comparative genomic hybridization study identified a maternally inherited hemizygous deletion of the Xp21 region of approximately 6.08 Mb that included both Duchenne muscular dystrophy and glycerol kinase genes, confirming the diagnosis. The patient was referred to metabolic and neurology consultations. Motor examination revealed a waddling gait when running, calf hypertrophy, and a positive Gower’s sign. Laboratory evaluation was notable for elevated creatine kinase, hyperglyceroluria, pseudohypertriglyceridemia, and increased transaminases. The patient had a normal adrenocorticotropic hormone stimulation test and normal aldosterone and renin levels. Currently, he has a multidisciplinary team follow-up, including therapies. He maintains deflazacort therapy and follows a nutrition plan based on a fat-restricted diet and avoidance of prolonged fasting to prevent further metabolic crises. This case highlights the importance of identifying the exact genetic defects, in addition to a global picture of symptoms. In our case, it was possible to diagnose complex kinase deficiency along with Duchenne muscular dystrophy. Consequently, it was optimal for multi-profile medical care accompanied by an adequate nutritional plan.

## Introduction

Contiguous gene deletion syndromes are disorders caused by deletions of large segments of genes that are adjacent to one another [1]. One of them is complex glycerol kinase deficiency (CGKD, OMIM: #300679, ORPHA: 261476), also called chromosome Xp21 deletion syndrome, a rare X-linked recessive disorder that is caused by microdeletion of genes located in Xp21 [2]. This region includes the genes responsible for glycerol kinase deficiency (GKD), X-linked adrenal hypoplasia congenita (AHC), Duchenne muscular dystrophy (DMD), chronic granulomatosis, ornithine carbamoyltransferase deficiency, and retinitis pigmentosa [1-3]. To diagnose CGKD, the presence of two of those genes in the deleted chromosome region is mandatory [2]. The AHC and DMD loci are the closest to the GKD, so the combination of their deletion is the most common genotype of CGKD [3,4]. Complex GKD with AHC usually presents in the neonatal period, but those who survive may present with failure to thrive, short stature, and genital alterations [4].

The clinical course of the disease depends on the size and range of the deletion, which contributes to the variability of clinical symptoms and therefore phenotypes [2,3,5]. More than 100 male patients have been reported so far [5]. Thus, rather than exhibiting a uniform disease course, patients with contiguous gene deletion syndromes present with historical, physical, and laboratory findings that depend on the specific genes affected, and treatment is directed toward each individual disorder (for example, GKD requires dietary management with regular intake of carbohydrate-rich meals and a fat-restricted diet) [5-7].

We aim to present a case of a Portuguese boy with Duchenne muscular dystrophy and GKD as part of the contiguous gene deletion syndrome Xp21, who was referred to our consultation. We also intend to describe his clinical evolution, highlight the critical role of genetics in the care and counseling of patients with this condition, and review the clinical features, pathogenesis, and treatment.

## Case presentation

We present the case of a Portuguese male, the only child of non-consanguineous parents with learning difficulties and a family history of low stature. The child was born from an uneventful full-term pregnancy with normal birth parameters (weight 3590 g; length 51 cm; Apgar score 10/10). In the first three years of life, our patient had two hospital admissions, one with hypoglycemia and vomiting at six months of age, and the other with metabolic acidosis during a febrile infection in the month after. Weight remained between the 3rd and 15th percentiles, stature around the 3rd percentile in the first three years of life, and then fell below this percentile thereafter. Regarding psychomotor development, our patient experienced a delay in the acquisition of language and autonomous gait, with these skills being acquired around 24 months. At three years of age, he continued to exhibit motor difficulties, including a wide gait, difficulty climbing stairs, and an inability to combine words, and was referred for neurodevelopmental consultation. Our patient was evaluated using the Schedule of Growing Skills, which indicated a developmental age of around 24 months in all areas except autonomy. The physical examination revealed strabismus and calf hypertrophy, and no dysmorphic features were noted. Later, our patient was referred for consultations with otorhinolaryngology, ophthalmology, and cardiology. Cardiac evaluation and hearing evaluation showed no alterations. Our patient started speech and psychomotor therapy with poor evolution, maintaining low scores at the Schedule of Growing Skills evaluation at four years and seven months of age, with few improvements, but no regression was observed.

**Figure 1:**
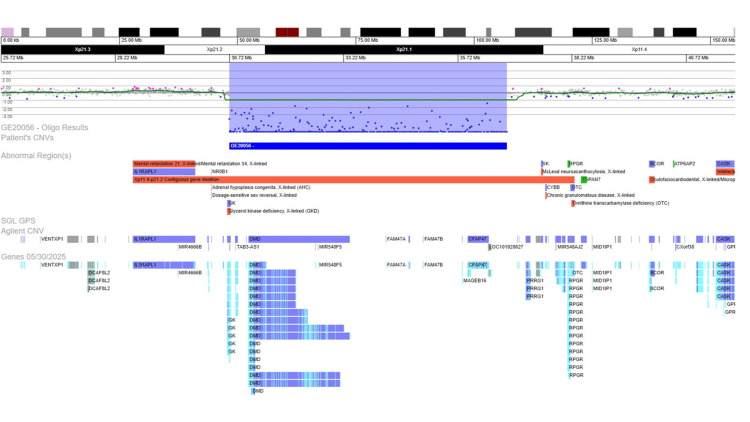
Array-comparative genomic hybridization (180k, Perkin-Elmer) on peripheral blood of the index case, showing a hemizygous Xp21.2-p21.1 deletion with approximately 6.08Mb including the entire DMD gene (*300377; #310200) and the terminal exons of all isoforms of GK (*300474; #307030)

**Figure 2:**
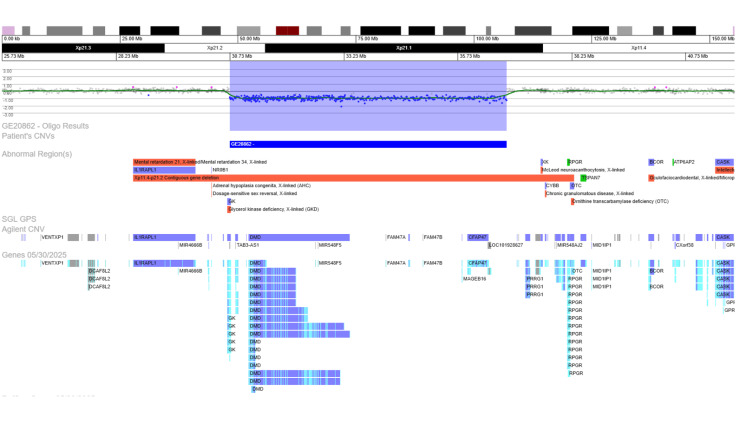
Array-comparative genomic hybridization (180k, Perkin-Elmer) on peripheral blood of the index case’s mother, showing the same Xp21.2-p21.1 deletion in a heterozygous state

The patient was referred for a genetic consultation, where an array-comparative genomic hybridization (array-CGH) on deoxyribonucleic acid extracted from peripheral blood was performed, which showed a hemizygous deletion with a size of approximately 6.08Mb in the region Xp21.2-p21.1 (Figure 1), affecting six genes, including the entire DMD gene (*300377; # 310200) and the terminal exons of all isoforms of GKD (*300474; # 307030) - arr[GRCh37] Xp21.2p21.1(30719577_36799314)x0. After additional studies, we confirmed that this deletion was also present in his mother, as seen in Figure 2.

**Figure 3:**
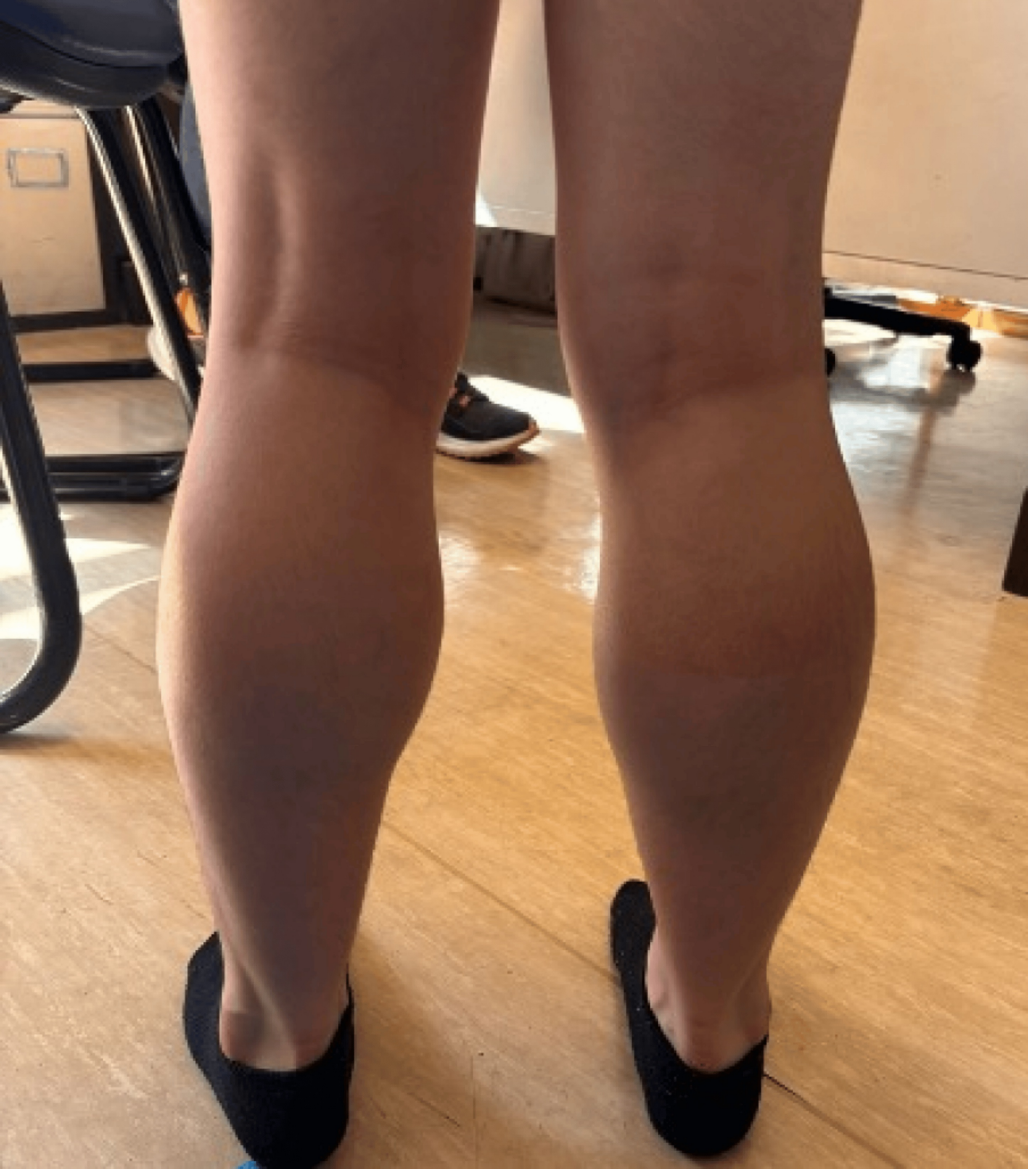
Calf pseudohypertrophy of our patient Informed consent was obtained.

The patient was referred to metabolic disease and neurology consults. Observation revealed calf pseudohypertrophy (as shown in Figure 3), proximal weakness in the lower limbs, Gowers’s sign, and myopathic gait with lumbar hyperlordosis. Laboratory results are presented in Table 1, showing an elevation in liver enzymes, serum creatine phosphokinase (CPK), and triglycerides (TG). The high concentration of serum TG without a turbid appearance of the serum sample led to the suspicion of pseudo-hypertriglyceridemia. The ionogram and aldosterone were normal. Urine organic acid analysis showed a prominent glycerol peak. The adrenocorticotropic hormone stimulation test was regular. Based on clinical, analytical, and genetic investigations, the patient was diagnosed with Duchenne muscular dystrophy and glycerol kinase deficiency. A tailored nutritional plan was initiated, which included a fat-restricted diet and avoidance of prolonged fasting. A month later, at five years and 11 months of age, and after ruling out adrenal insufficiency, treatment with deflazacort 0.9 mg/Kg/day and vitamin D supplementation was started.

**Table 1:** Laboratory results of the patient

	Patient's result	Normal range
Aspartate aminotransferase	207 U/L	0-40 U/L
Alanine transaminase	443 U/L	0-41 U/L
Creatine phosphokinase (CPK)	12 275 - 15 800 U/L	30.160 U/L
Fasting triglycerides	373 mg/dL	< 150 mg/dL

The patient maintains a multidisciplinary follow-up across several specialities, including neurology, metabolic diseases, physical medicine and rehabilitation, neurodevelopment, endocrinology, ophthalmology, pneumology, and cardiology. Support from occupational, speech, and psychomotor therapies has also been provided. No cases of adrenal crisis have been observed to date. There were no further metabolic crises.

## Discussion

**Figure 4:**
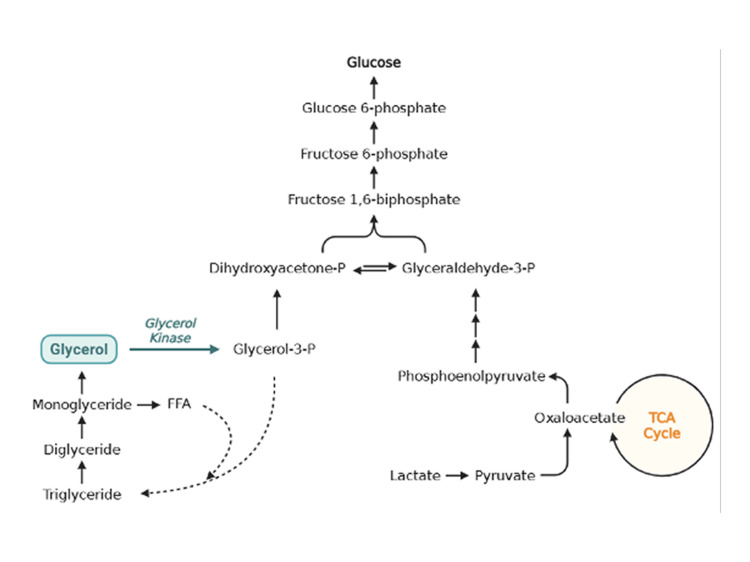
Expanded view of the glycerol pathway Due to the absence of glycerol kinase, glycerol is not phosphorylated into glycerol-3-phosphate, which affects gluconeogenesis and can lead to hypoglycemia. In contrast, glycerol accumulates in the blood and urine (detected as pseudohypertriglyceridemia) and is diverted to glycolysis, increasing lactate and pyruvate levels, contributing to metabolic acidosis. Created on https://BioRender.com

The Xp21 contiguous gene deletion syndrome is a rare disorder that can affect multiple genes. Glycerol kinase is the enzyme responsible for the phosphorylation of glycerol from triglyceride breakdown, a precursor for gluconeogenesis or the esterification of fatty acids [7], as seen in Figure 4. The absence of this enzyme leads to the accumulation of glycerol in circulation, causing glycerolemia, which is usually detected as pseudohypertriglyceridemia due to analytical interference by free glycerol and glyceroluria, as in this case [7-9].

Glycerol kinase deficiency is an X-linked recessive disorder that can occur in an isolated or complex form, as shown by this case [7]. It can also lead to hypoglycemia due to its impact on gluconeogenesis; however, this mechanism of glycerol appears to be more important for energy production in the child’s brain compared to that of an adult, which can explain why symptoms are less intense and frequent during adulthood than in infantile forms [7].

Disruption of the GK gene can produce different phenotypes, ranging from asymptomatic hyperglycerolaemia and pseudohypertriglyceridemia, as this case presents, to a severe metabolic crisis with vomiting, hypoglycemia, loss of consciousness, and seizures during episodes of increased catabolism [10]. It can also be accompanied by growth retardation, delayed psychomotor development, and bone dysplasia in childhood [10].

As previously reported, a whole DMD gene deletion was present in this case [2]. There was no diagnosis of primary adrenal insufficiency, which is consistent with the absence of NR0B1 deletion. Despite not having affection for the IL1RAPL gene, which is responsible for intellectual disability and developmental delay, intellectual disability can have a heterogeneous cause and occur because of the deletion of the DMD gene, as it seems in this case [1,2,5]. The lifetime of males affected by CGKD is often shortened [2]. The first critical period is infancy or early childhood because of the manifestation of adrenal dysfunction, if NR0B1 is involved, which might be misdiagnosed.

In most cases, CGKD occurs de novo; however, inheritance from clinically healthy carrier mothers can also occur, as shown by this case [9,10]. Symptoms are present mainly in males because CGKD is inherited in an X-linked recessive mode [10]. Sometimes, female carriers may also have symptoms (intellectual disability and inconstant mild muscular symptoms) if skewed X-chromosome inactivation occurs, which can explain the mother’s learning difficulties [11]. The molecular diagnosis of CGKD is also essential to allow genetic counselling for the family, namely, regarding the risk of recurrence in future siblings of the proband and reproductive options for the parents (invasive prenatal diagnosis or preimplantation genetic testing), identification of other at-risk family members, and clinical management of other affected and carrier relatives [5]. In the case of asymptomatic carriers’ family members, as the mother, it is important to arrange a cardiology consult, since heterozygotes for DMD have an increased risk for cardiomyopathy [12].

A development delay in the presence of GKD should make us suspect CGKD, as it is more commonly seen in a complex form rather than isolated GKD [3,11]. Early diagnosis of CGKD allows for the optimization of medical care and follow-up, which positively impacts individual development and quality of life [6,8]. The overall prognosis of this disease is gene-dependent. As for this patient, with deletions affecting both the DMD and GK genes, the long-term outcome is primarily determined by the course of Duchenne muscular dystrophy, which dictates progressive muscle weakness and potential cardiac and respiratory complications. Multidisciplinary management with the early use of corticosteroids and physiotherapy can optimize mobility, muscle strength, and quality of life.

## Conclusions

In summary, this case reinforces the value of early genetic testing to diagnose diseases that are not linked, such as a contiguous gene deletion syndrome, and it also contributes to counseling the patient and his family. To our knowledge, no cases of CGKD have been reported in the Portuguese literature, emphasizing the importance of pursuing an etiologic diagnosis in the presence of severe global developmental delay with alarming signs. A limitation of the present study is its single-case design. Due to the extreme rarity of contiguous gene deletion syndromes, it is challenging to collect more cases or extract further information, which limits the generalizability of the findings.
